# Electropulse‐Driven Dislocation Evolution in Sub‐10 nm Metallic Nanocrystals

**DOI:** 10.1002/advs.77124

**Published:** 2026-08-12

**Authors:** Youran Hong, Xiaoyu Zhai, Xing Li, Xinfang Zhang, Kexing Song, Ze Zhang, Jiangwei Wang

**Affiliations:** ^1^ Center of Electron Microscopy State Key Laboratory of Silicon and Advanced Semiconductor Materials School of Materials Science and Engineering Zhejiang University Hangzhou People's Republic of China; ^2^ Key Laboratory of Green Extraction and Efficient Utilization of Light Rare‐Earth Resources Inner Mongolia University of Science and Technology Baotou People's Republic of China; ^3^ Institute of Materials Henan Academy of Sciences Zhengzhou People's Republic of China

**Keywords:** dislocation evolution, electroplasticity, structural heterogeneity, sub‐10 nm interconnects

## Abstract

As critical microelectronic components are scaled down to the sub‐10 nm level, they experience extreme current densities that inevitably trigger defect formation and evolution. Understanding dislocation dynamics and electro‐induced damage at this particular scale is therefore crucial, as it governs the reliability of next‐generation nanodevices. Herein, we investigate the dislocation evolution in Mo and Pt microcrystals upon pulse stimulation. By tracking dislocation generation, motion, and annihilation pulse‐by‐pulse, we reveal that enhanced electron–lattice interactions induce dislocation nucleation from sites of structural heterogeneity, in the form of dislocation loops. These dislocations experience frequent interaction and annihilation in the subsequent electropulsing process, inducing a periodic variation of dislocation density and contributing to the structural disordering. These findings not only provide insights into the structural degradation of metallic nano‐interconnects during service but also have important implications for understanding the electroplasticity in bulk materials.

## Graphical abstract

Atomic‐scale TEM imaging reveals how nanosecond electropulses drive dislocation evolution in sub‐10 nm metallic nanocrystals. Enhanced electron scattering at structural heterogeneities nucleates dislocation loops and dipoles, which then repeatedly interact and annihilate, causing dislocation density fluctuations or structural disordering. These observations offer atomic insights into the reliability of nanoscale interconnects and the fundamentals of electroplasticity.

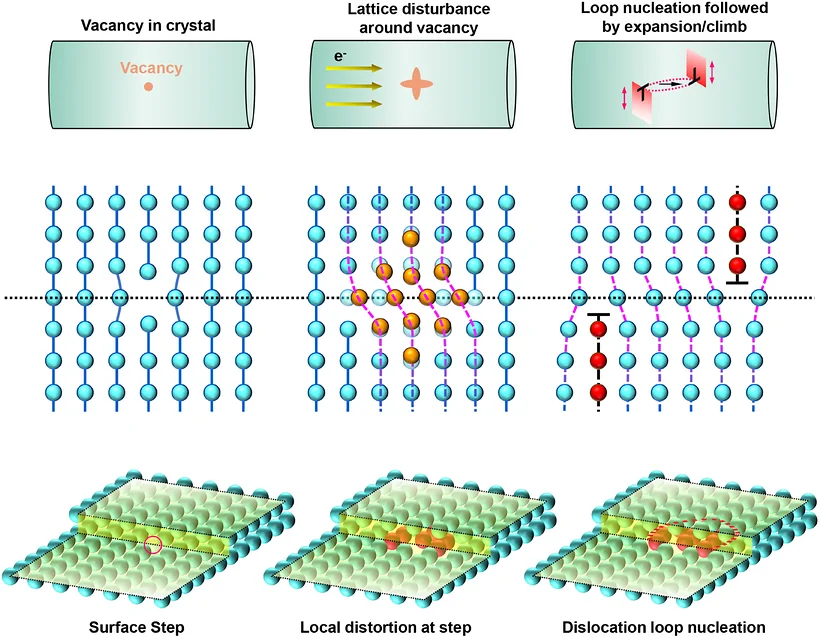

## 1 Introduction

Fast developments of artificial intelligence, big data and supercomputing, next‐generation high‐power devices, etc. eagerly desire advanced processors, data storage equipment and microelectromechanical systems (MEMS) with smaller feature sizes, ultra‐high operational frequencies and higher reliability [1, 2, 3, 4]. Currently, miniaturization of nanoelectronics has pushed the envelope of their metallic nanocomponents down to the atomic scale [4, 5, 6]. With the size reduction, current density passing through these nanocomponents increases markedly, inevitably inducing defect generation and structural variation under electrical stimuli [7]. Given the limited volume of nanocomponents, the generation and evolution of individual defects can significantly influence their structural stability and functional properties [8, 9, 10]. For example, the formation of individual twin boundaries can influence the resistivity of Mo nanowires [11]. Thus, a fundamental understanding of defect dynamics under electric fields is important for the reliability of micro/nano‐electronics.

As the primary plastic carriers in crystalline materials, dislocations play a crucial role in governing materials’ mechanical and functional performances. Numerous investigations have shown that dislocation dynamics under electrical stimuli is particularly critical for electroplasticity, electromigration‐induced damage, and ultimately, the structural failure of electronic components [12, 13, 14, 15]. Although significant research has elucidated the influence of electric currents (e.g., via electron wind force) on dislocation generation, structural evolution (including defect multiplication, interaction, and annihilation, etc.), and resultant property variations in bulk materials [14, 15, 16], the fundamental mechanisms governing dislocation dynamics remain largely underexplored, due to the complex dislocation interactions. This issue becomes especially pronounced and scientifically intriguing at the sub‐10 nm regime. In such a confined volume, enhanced current density may easily impact the defect evolution, while high image force can also change dislocation behaviors due to the easy dislocation escape to the free surface [17]. This intrinsic scarcity of dislocations renders sub‐10 nm nanocrystals an exceptionally suitable platform for isolating and investigating the fundamental processes of individual dislocations, by eliminating the complex interactions within dense dislocation networks. This understanding is not only important for the reliability of micro/nano‐electronics but also can inspire the development of electric‐assisted manufacturing and processes for materials with poor formability.

Nowadays, dynamic transmission electron microscopy has enabled real‐time imaging of transient processes with nanosecond resolution [18, 19, 20], albeit at the cost of spatial resolution, which makes atomic‐scale observations in sub‐10 nm nanostructures challenging. Here, we directly visualize the dislocation evolution in metallic nanowires subjected to nanosecond electropulse, using an integrated in situ nanofabrication‐electropulsing testing in high‐resolution transmission electron microscopy (HRTEM). It shows that electropulsing induces severe lattice distortion and structural heterogeneity, which finally results in dislocation nucleation in the form of dipoles. Pulse‐induced dislocations are subject to dynamic recovery via dislocation annihilation and regeneration, contributing to a periodic fluctuation of dislocation density. This pulse‐induced dislocation evolution offers atomistic insights into the structural degradation in metallic nanointerconnects, and holds important implications for the electroplasticity and pulse‐assisted forming of metals and alloys.

## 2 Results and Discussion

Transition metal Mo, as an alternative interconnect material for next‐generation nanochips [21, 22], is used as the model system in this study. During experiments, Mo bicrystal nano‐interconnects were fabricated by an in situ nanowelding method (see Methods), and then subjected to the electropulse application to study the defect behaviors. Figure 1 presents the sequential HRTEM images showing the representative processes of dislocation evolution in a Mo nanowire. The as‐welded Mo bicrystal nanowire has a diameter of 10.3 nm and contains a high‐angle grain boundary (GB), as shown in Figure 1a. In the pristine sample, few edge dislocations (Figure S1) are observed in both grains of the nanowire. Subsequently, individual electropulses are applied sequentially to directly visualize the dislocation evolution at the atomic scale. As shown in Figure 1b, the application of merely one electropulse would disturb the initial lattice and induce the generation of both lattice distortion (in the left grain, see the inverse fast Fourier transform (IFFT) in Figure 1e) and dislocation dipoles (in the right grain) that contain two dislocations with opposite signs. The lattice distortion is a kind of symmetry‐breaking in the crystal, which can act as a precursor for further nucleation of dislocation dipoles, as evidenced by the formation of a new dislocation dipole in the left grain after the application of the second electropulse (Figure 1c and the IFFT image in Figure 1f). Note that the position of this dislocation dipole deviates slightly from the pre‐formed lattice distortion, which can be ascribed to the simultaneous dislocation motion after nucleation. In the meantime, dislocation dipoles in the right grains annihilate after the second electropulse, validating the inference of dislocation motion after nucleation. With further application of electropulses, more dislocation dipoles generate, move, and annihilate dynamically (Figure 1d). Besides the dislocation dipoles, few individual dislocations are observed to nucleate near the surface region or at the surface step (marked by D_1_ and D_2_ in Figure 1c), and show similar annihilation behavior in subsequent electropulses. Besides, the configuration of pre‐existing GB also shows some variations but has limit impact on the dislocation behaviors, which will not be discussed here.

**FIGURE 1:**
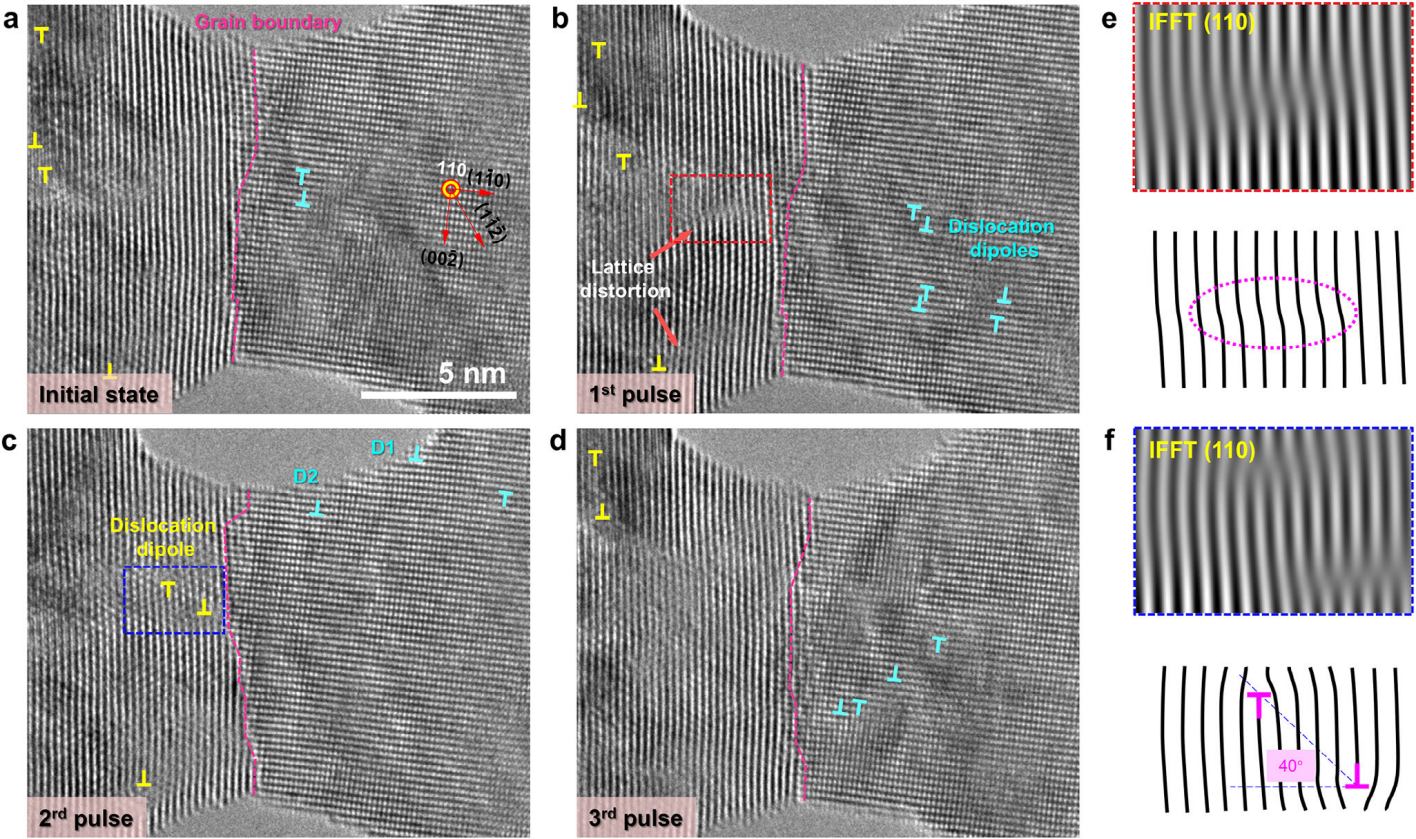
Atomic‐scale observation of electropulse‐driven dislocation evolution in Mo bicrystal nanowire. (a) Initial state of an as‐welded nanowire of 10.3 nm diameter containing a high‐angle grain boundary (GB). Edge dislocations are marked by “T”. (b) The first pulse induces lattice distortion in the left grain and dislocation dipoles in the right grain. (c) The second pulse triggers the nucleation of a new dislocation dipole from the distorted region in the left grain, while the initial dipole in the right grain annihilates. Individual dislocations (D1, D2) nucleate near the surface. (d) The third pulse leads to further generation, motion, and annihilation of dislocations. (e,f) IFFT images of the areas outlined in (b,c), highlighting the lattice distortion and the core structure of the newly nucleated dislocation dipole, respectively.

The electropulsing‐induced lattice distortion and dipole dynamics are common in bicrystal Mo nanowires with different diameters and crystallographic orientations, under different pulse parameters. Figure 2a further quantifies the statistical dislocation behaviors in three Mo nanowires (Figures S2–S4) under the sequentially applied electropulses. In these nanowires, different numbers of dislocations preexist; with the electropulsing, the numbers of dislocations show a gradual increase, during which nucleation, rearrangement, and annihilation of dislocation arrays evolve dynamically and competitively in the nanowires, like the processes presented in Figure 1. At a high level of dislocation density, further applications of electropulse would induce a net decrease of the dislocation density. This sharp reduction should originate from two aspects: (i) high density of dislocations enhances the probability of interaction between each other and induces self‐annihilation, either in the same dipole or between neighboring ones; (ii) the small volume of nanowire samples can promote fast surface annihilation due to the large surface image force and dislocation starvation effect [17]. Such dislocation saturation–annihilation process occurs repeatedly with the application of electropulses, even after more than 100 pulses (Figure S5). These observations also suggest that to some extent, the dislocation density in small‐volume crystals can be modulated by the application of electropulses, similar to the pulse‐induced plasticity and structural evolution in bulk materials.

**FIGURE 2:**
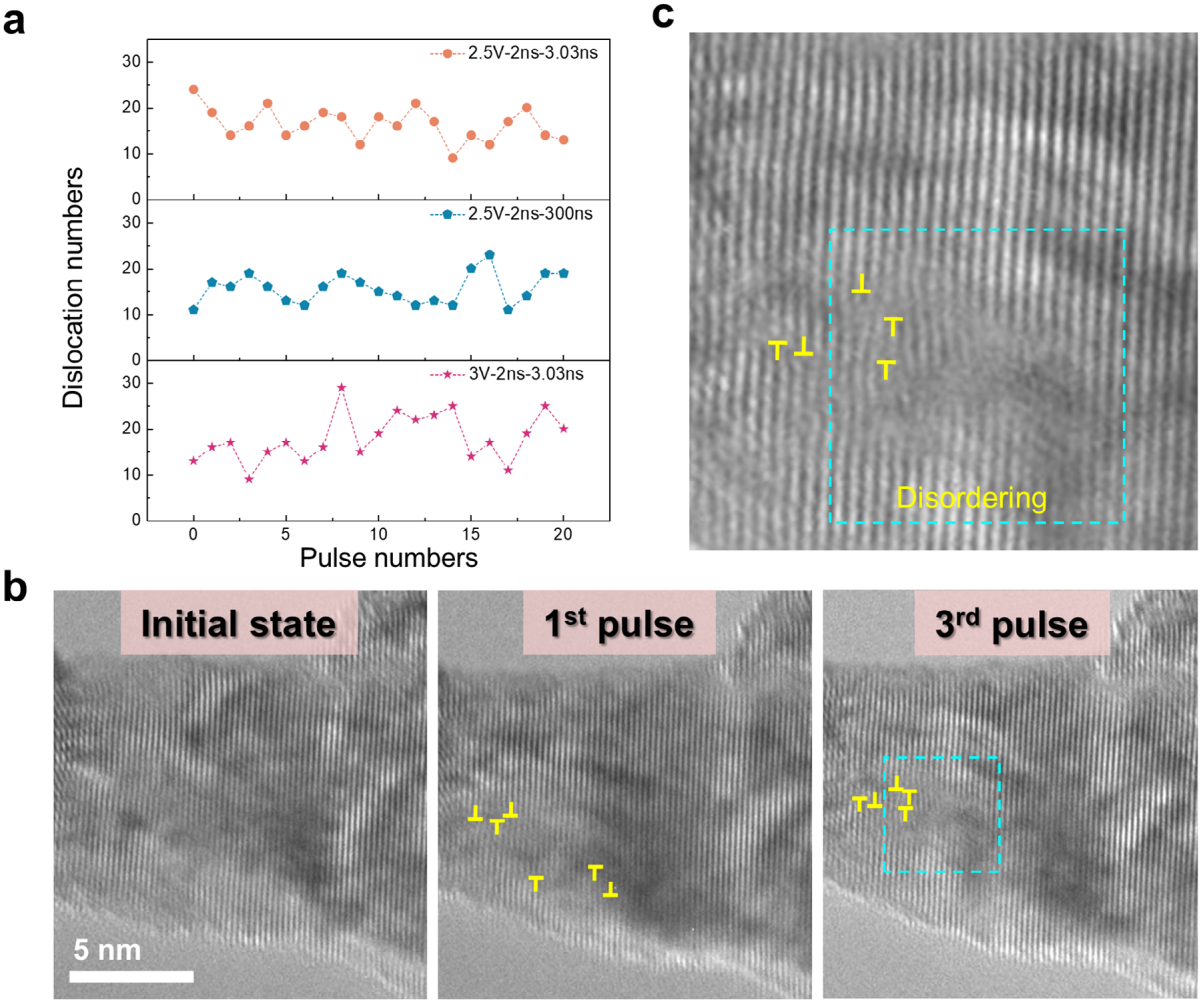
Structural evolution induced by electropulsing in Mo nanowires. (a) Statistical evolution of dislocation density in three individual nanowires under sequential electropulsing. (b) Localized disordering formation under the sequentially applied pulses. An initial electropulse introduces dislocations, and subsequent pulses induce lattice distortions to accumulate and yield a localized disordered region (outlined by the cyan dashed rectangle). (c) Enlarged image of the enclosed area in (b).

More intriguingly, severe development of electropulse‐induced lattice distortion and/or dislocation interaction can result in the generation of localized disordering. As shown in Figure 2b, application of the first pulse induces several dislocations in the initial lattice. The as‐formed dislocations break the symmetry of the crystal lattice, which increases the localized scattering of electrons and thereby enhances the lattice–electron interactions at the dislocation cores. In the subsequent process, the pulse‐induced lattice distortions tend to further develop near the core of pre‐generated dislocations; in the meantime, the pre‐generated dislocations can also interact with each other upon the application of further electropulses. Both processes can contribute to the accumulation of localized lattice disturbance, which, at some point, would induce the formation of localized disordering nearby the pre‐formed dislocations, as shown in Figure 2c.

Theoretically, the pulse‐induced lattice distortion, dislocation generation, and lattice disordering should involve the electron–lattice interactions (i.e., electron–phonon coupling), where the structural heterogeneities likely play significant roles by locally enhancing electron scattering. At such defect sites, the energy and momentum transfer from drifting electrons can concentrate locally and drive atomic rearrangements. In our experiments, dislocation dipoles are frequently observed in the electropulsed Mo nanowires, and these dislocation dipoles are actually half dislocation loops expanding on the {110} planes [23]. Nucleation of these dislocation dipoles may involve two possibilities, as schematically illustrated in Figure 3. Typically, structural heterogeneities in materials tend to act as hotspots to increase the localized electron scattering, resulting in enhanced defect mobility during electropulsing [24]. For the region without a lattice defect, a vacancy may act as a structural heterogeneity to increase the localized electron–phonon interactions under electropulse. This would induce exaggerated atomic vibrations, which, in conjunction with the extra space provided by a pre‐existing vacancy for atom rearrangement, can deviate atoms from their equilibrium sites, shown as localized lattice distortion (see the schematic in Figure 3a,b, from both global and atomic perspectives). These lattice distortions further intensify localized electron–phonon interactions and thus generate atomic‐scale shear strains near the vacancy site [13], triggering the homogeneous nucleation of a dislocation loop in subsequent electropulsing (shown as dipoles in the projective image). The processes in Figure 1b,c validate the above mechanism of dislocation nucleation. We also notice that sometimes the core of dislocation dipoles shows a deviation from the site of pre‐formed lattice distortion (see Figure 1c). This is because the as‐nucleated dislocation loop would experience some expansion on its slip plane ({110} planes for Mo) or climb perpendicularly after the nucleation, to relax the residual stress [24]. In subsequent electropulsing, lattice vibration is more active at the dislocation core because of the electron–defect interaction, resulting in further motion of dislocation dipoles and final annihilation with each other or toward the free surface (shown as the dynamic evolution of dislocation density in Figure 2a). Note that although Joule heating is largely eliminated by the application of nanosecond pulse (Note S1), it can facilitate dislocation nucleation by reducing the energy barrier. Nucleation of dislocation dipoles (with opposite signs) also suggests non‐directional electron–defect interactions, consistent with the previous study [24].

**FIGURE 3:**
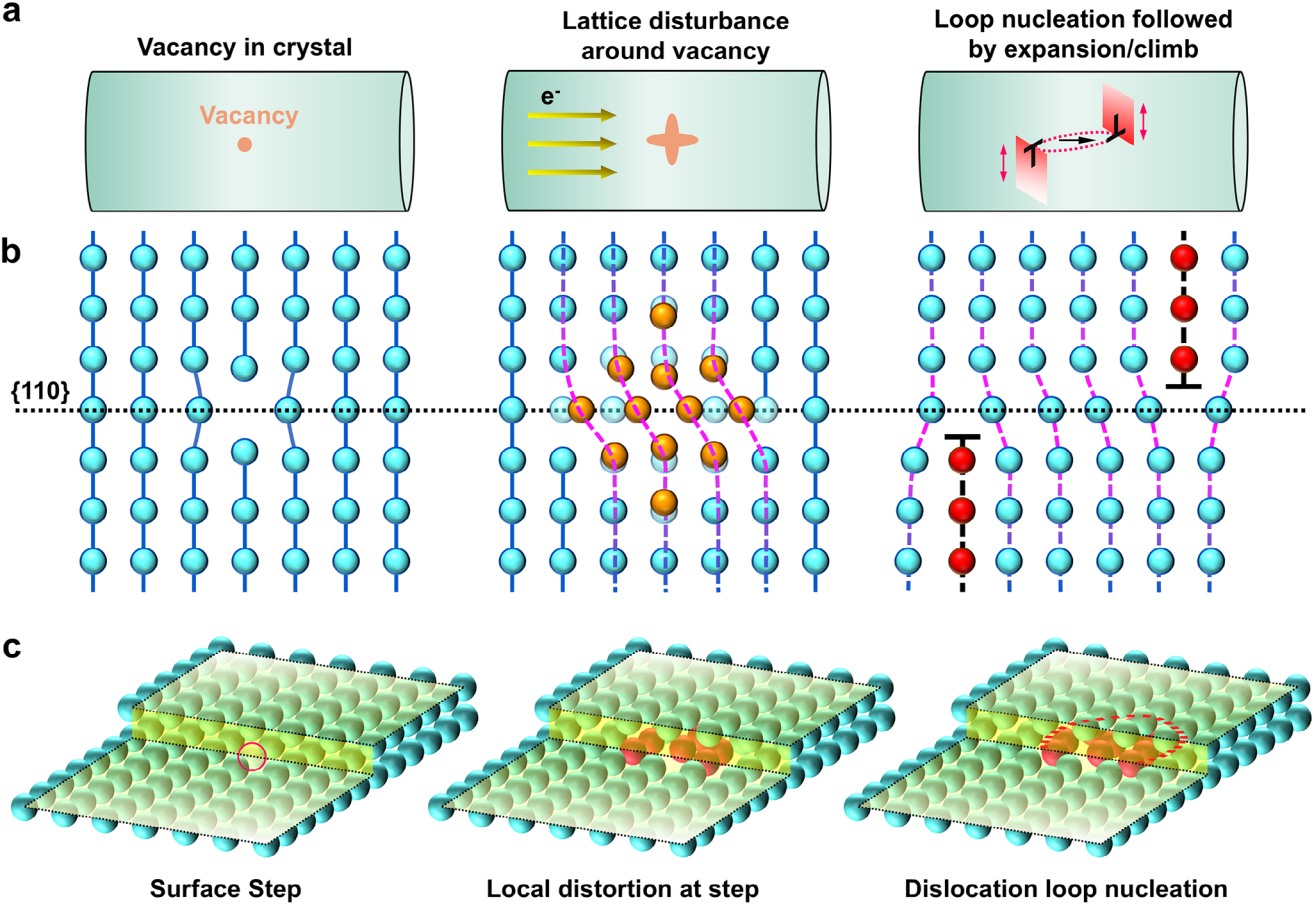
Schematic showing electropulse‐induced dislocation nucleation and evolution in Mo nanowires. (a,b) Schematic illustration of the dislocation loop nucleation mechanism via a vacancy. The activated atoms around the vacancy are shown by the orange balls in (b), and the red balls indicate the core of the dislocation. The newly formed dislocation loop can expand on the {110} slip plane (represented by the black dotted line) or climb to relax stress. (c) Dislocation nucleation from a surface step as a heterogeneous site.

Surface steps can also act as the heterogeneous sites for stress concentration, favoring the nucleation of dislocation loops [23, 25]. As schematically shown in Figure 3c, enhanced electron scattering at surface step sites can induce localized stress concentration and lattice distortion under pulse conditions [26]. As the distortion develops, a half dislocation loop can be emitted from the surface step site. The nucleation of dislocations D_1_ and D_2_ in Figure 1c directly demonstrates this nucleation mechanism. After nucleation, these dislocations can either propagate into the crystal or annihilate at the free surface in subsequent pulsing (see Figure 1d), contributing to the dynamic variation of dislocation density. This surface nucleation mechanism is further validated by an in situ observation in a Pt nanocrystal (Figure 4). In Figure 4a, two nanograins with minor crystallographic mismatch (∼12°) were welded together, forming a bent coherent nanocontact viewed along the [01$\bar{1}$] zone axis. Upon applying a single electropulse (2 V for 5 ns), four new atom columns emerge on the tensile surface of the nanocontact, constituting a surface dislocation core near the surface step (Figure 4b). Subsequent application of the second pulse triggers dislocation climb accompanied by elimination of a row of atomic columns (Figure 4c) as well as surface adatom columns. Both the dislocation nucleation and climb processes involve surface atom migration, evidenced by the fluctuating number of adatom rows at the nanocontact surface in Figure 4a–c, indicating that the current promotes atomic rearrangement both on the surface and around the dislocation core. The third pulse induces annihilation of the previously observed dislocation through recovery of the missing atomic row, while simultaneously generating a new surface dislocation on the tensile side (Figure 4d). These pulse‐by‐pulse observations suggest that electromigration‐driven surface atom diffusion should have some contributions to the dislocation nucleation from surface sites and their subsequent climb, by supplying atoms for the formation and growth of an extra atomic plane at the dislocation core.

**FIGURE 4:**
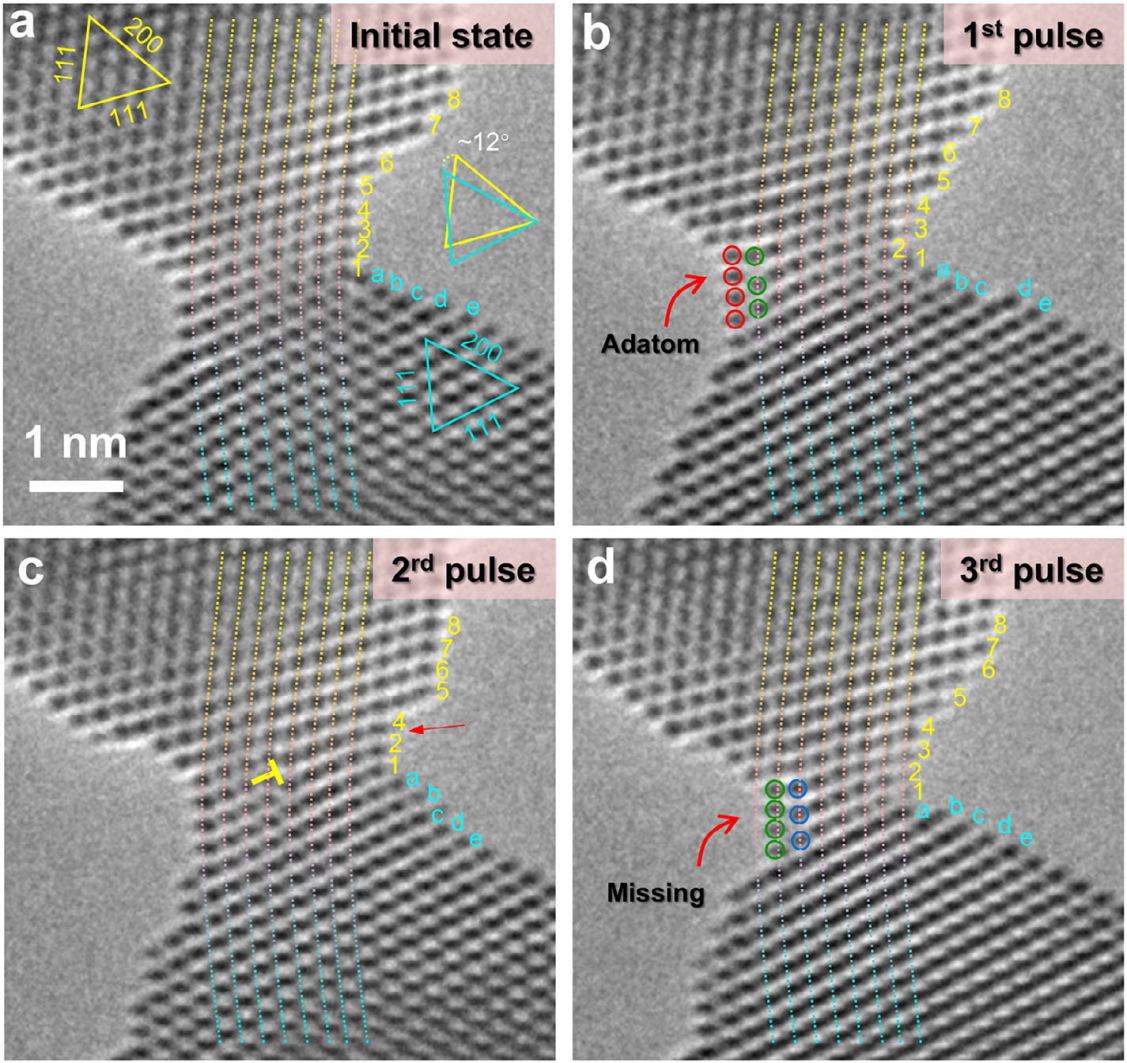
Direct observation of dislocation nucleation and evolution at Pt nanocontact surface. (a) Initial bent coherent nanocontact formed by welding two nanograins with a ∼12° mismatch viewed along the [01$\bar{1}$] zone axis. The coherent {111} planes are marked by eight gradient‐colored dotted lines as reference. (b) A single electropulse (2 V, 5 ns) induces four new adatom columns on the tensile side near a surface step, constituting a surface dislocation core. (c) A second pulse drives dislocation climb, accompanied by the elimination of an adjacent row of atomic columns (marked by the red arrow) and surface adatom columns. (d) A third pulse leads to dislocation annihilation through atomic row recovery, while nucleating a new surface dislocation on the tensile side.

The above observations directly visualize the atomistic dislocation evolution upon electropulse. These dislocation behaviors not only constitute a major form of structural degradation of nano‐interconnect during service, but also represent the typical dislocation dynamics during the electroplasticity of bulk materials. For metallic nano‐interconnects, pulse‐induced lattice distortion and dislocation increase the electron scattering and thereby enhance the resistivity [27]. Severe development of electron–defect interactions can even induce localized disordering in the lattice, further degrading the service life of nanosized components [15, 28]. Surface‐mediated dislocation nucleation and surface electromigration are also important forms of degradation that destroy the structural integrity of metallic nanocrystals [29, 30], while the electropulse‐assisted dislocation climb and annihilation may contribute to the structural relaxation in nanosized crystals [31, 32]. All these processes can impair the functional properties and structural stability of metallic nano‐interconnects in subsequent working processes. These understandings of dislocation evolutions also provide an opportunity for the atomistic manufacture of metallic nanocrystals via defect engineering regulated by electropulsing.

Further, dislocation density in metals shows dynamic fluctuations with increasing pulse numbers (Figure 2a), suggesting that electropulsing not only provides the driving force for dislocation nucleation but also facilitates their annihilation and reorganization. This dislocation dynamics can be closely correlated with the dynamic softening–hardening observed in the electroplasticity of different metals and alloys [33, 34]. Typically, intense electron–defect interaction promotes the dynamic nucleation, redistribution, interaction, and annihilation of both vacancies and dislocations, thereby preventing severe accumulation of stored energy and work hardening [35]. Extended pulsing experiments (Figures S5 and S6 and Notes S2 and S3) further confirm that dynamic fluctuation in dislocation density persists even after prolonged pulsing, although the dislocation density shows a gradual decrease over 200 pulses due to progressive source exhaustion and structural relaxation. This cyclic process of defect generation and annihilation under electropulsing shares a remarkable similarity with the early stages of dynamic recrystallization during thermo‐mechanical processing [36], where the nucleation and movement of dislocations are driven by the stored energy in the deformation and the outer energy (heat). While, in the electropulsing‐assisted deformation, the drift electrons can interact directly with dislocations and improve their mobility, which can greatly accelerate the dynamic recrystallization [37, 38]. Our in situ observations reveal the atomistic evolution of dislocations during electroplasticity, which holds significant implications for understanding the pulse‐assisted workability enhancement and stress relaxation of bulk materials at macroscale [39, 40, 41]. Besides, electropulse‐enhanced lattice vibrations create additional phonon drag to suppress dislocation glide during electroplasticity [42], such that intense dislocation interactions may occur in localized regions, contributing to the structural disordering (Figure 2b,c). This provides a possible atomistic origin for pulse‐induced amorphization in crystals [28, 43, 44]. Last, it needs to be noted that GBs are also primary sites for electron scattering, which would suffer from migration and dislocation emission under an electrical field [45]. With the intense electron scattering, GB can also act as the initiation site for amorphization due to the increased atomic disordering [46, 47]. In current experiments, some structural variations and lattice distortions were observed near the GB of a bicrystal nanowire, and dislocation nucleation was captured near the GB. Pulse‐enhanced lattice vibrations at the GB should have some contributions to these processes in our experiments.

## 3 Conclusion

In metallic nanomaterials, the interaction of electric current and dislocations is often considered a key factor contributing to electroplasticity and structural degradation. In this article, we provide direct atomistic visualization of dislocation evolution in a metallic nanocrystal under the electropulse condition. Enhanced electron–phonon interactions induce dislocation nucleation from structural heterogeneities, in the form of dislocation loops. Further electropulsing induces a periodic variation of dislocation density, where dynamic interaction and annihilation proceed frequently, contributing to the structural disordering and degradation. These findings not only provide insights into the structural degradation during the service of metallic nano‐interconnects, but also have important implications for understanding the electroplasticity in bulk materials.

## 4 Experimental Section

### 4.1 Sample Preparation and Characterization

The in situ fabrication of Mo and Pt nanocrystals was carried out inside a Cs‐corrected FEI G2 60–300 TEM (operated at 300 kV) using a Pico‐Femto TEM electromechanical holder (Zeptool Co.). Since the preparation methods of Mo and Pt nanocrystals are basically the same, here we will only describe the preparation of Mo nanocrystals. First, two bulk Mo rods (0.25 mm in diameter, 99.9 wt.%, Alfa Aesar Inc.) were ultrasonically cleaned in ethyl alcohol to minimize possible contamination. Then, the cleaned rods were cut by a wire cutter to get fresh and clean surfaces with numerous tips in nanoscale. One rod was loaded onto the probe side used as a scanning tunneling microscope (STM) probe of the TEM holder, and the other rod was loaded onto the static side. Before ever in situ nanofabrication of nanocrystals, the nanotips on the fracture surface of Mo rods with appropriate zone axis were searched to obtain the specific grain boundaries and enable the atomic resolution for in situ testing. After searching, the Mo rods on the STM probe were driven by a piezo‐controller to approach the rod on the static side to achieve a point contact with each other. Then, a square electropulse was applied by a pulse generator (Keysight, 81110A configured with one output module “Agilent 81111A 165 MHz 10 V”), which welded the two nanotips together, forming two nanocrystals with one grain boundary. The pulse parameters were set as 2.5–3.0 V (the pulse amplitude) – 2.0 ns (the transition time, defined as the interval between the 10%‐ and 90%‐ amplitude points on the leading/trailing edge) – 3.03–300 ns (the pulse width, defined as the interval between leading‐ and trailing‐edge medians). As for in situ electropulsing experiments, individual pulses were applied sequentially on the nanocrystals, resulting in the nucleation, migration, and annihilation of dislocations. During electropulsing experiments, no external mechanical loading was applied or pre‐applied. A Gatan 994 charge‐coupled device (CCD) camera was used to capture the evolution of dislocations in real time (∼0.3 s per frame) during experiments.

### 4.2 Dislocation Counting

To track dislocation evolution under sequential electropulses, we manually counted the total number of dislocations in one of the two grains of the bicrystal naonowire (as shown in Figures S2–S4), using HRTEM images. The counting criteria are as followed: (i) a dislocation is identified by an extra half‐plane of lattice fringes in HRTEM images, typically after inverse fast Fourier transform treatment to enhance the specific lattice planes; (ii) only dislocations clearly resolvable in the nanowire are counted, excluding those obscured by surface contamination or overlapping with GBs; (iii) dislocation dipoles are counted as two individual dislocations. Since the nanowire volume remains approximately constant during each short pulse sequence, the absolute dislocation count can be directly used as a measure of relative dislocation density.

## Author Contributions

**Youran Hong**: investigation, validation, formal analysis, visualization, Writing – original draft. **Xiaoyu Zhai**: investigation, formal analysis, validation. **Xing Li**: investigation, formal analysis, writing – review and editing. **Xinfang Zhang**: writing – review and editing, funding acquisition. **Kexing Song**: writing – review and editing. **Ze Zhang**: resources, writing – review and editing. **Jiangwei Wang**: conceptualization, methodology, funding acquisition, project administration, resources, supervision, data curation, validation, writing – review and editing.

## Funding

This work was supported by the Zhejiang Provincial Natural Science Foundation of China (LR24E010002), the Startup Research Fund of Henan Academy of Sciences (232017011), and the National Natural Science Foundation of China (52474410).

## Conflicts of Interest

The authors declare no conflicts of interest.

## Supporting information




**Supporting File**: advs77124‐sup‐0001‐SuppMat.docx.

## Data Availability

The data that support the findings of this study are available from the corresponding author upon request.
