# Supplementary material for: Electropulse‐Driven Dislocation Evolution in Sub‐10 nm Metallic Nanocrystals
Source: Adv Sci (Weinh). 2026 Aug 12:e77124. Online ahead of print. doi: 10.1002/advs.77124 (PMC13464551; doi:10.1002/advs.77124)
Supplement: Supplementary file 1 [file ADVS-9999-e77124-s001.docx]

Supplementary Information

**Electropulse-driven dislocation evolution in sub-10-nm metallic nanocrystals**

*Youran Hong^#^, Xiaoyu Zhai^#^, Xing Li^#^, Xinfang Zhang, Kexing Song, Ze Zhang, Jiangwei Wang**

Figures S1-S8

Note S1-S2

Reference 48-50

**Figure S1. High resolution TEM images that show dislocations with edge components in Mo and Pt nanocrystals.** (a) and (b) correspond to edge dislocations in the Mo nanocrystals from Figure 1. (c) corresponds to edge dislocation in the Pt nanocrystal from Figure 4. Scale bars are 1 nm.

**Figure S2. Structural evolution induced by electropulsing in Mo nanowire under (2.5 V-2 ns- 3.03 ns) electropulsing.** (a) The initial state. (b) After 10 pulses. (c) After 20 pulses. (d) Statistical evolution of edge dislocation (ED) numbers in the right nanocrystal, where edge dislocations with the extra half-plane of atoms oriented upward are defined as positive (the same below). The quantities of positive and negative dislocations remain nearly identical throughout.

**Figure S3. Structural evolution induced by electropulsing in Mo nanowire under (2.5 V-2 ns- 3000 ns) electropulsing.** (a) The initial state. (b) After 10 pulses. (c) After 20 pulses. (d) Statistical evolution of ED numbers in the right nanocrystal. The quantities of positive and negative dislocations remain nearly identical throughout.

**Figure S4. Structural evolution induced by electropulsing in Mo nanowire under (3 V-2 ns- 3.03ns) electropulsing.** (a) The initial state. (b) After 10 pulses. (c) After 20 pulses. (d) Statistical evolution of ED numbers in the right nanocrystal. The quantities of positive and negative dislocations remain nearly identical throughout.

**Figure S5. Long-term structural evolution induced by electropulsing in a Mo nanowire (2.5 V-2 ns- 1500 ns).** (a) Statistical evolution of edge dislocation number and grain boundary (GB) width in the nanowire under sequential electropulsing. (b) The HRTEM images of the nanowire at initial state, after 100 pulses and after 200 pulses.

**Figure S6. Long-term structural evolution induced by electropulsing under different voltages in the Mo nanowire.** (a) Statistical evolution of edge dislocation number and GB width in the nanowire under sequential electropulsing. (b) The HRTEM images of the nanowire after 90, 120 and 200 pulses, respectively.

**Figure S7. Statistical evolution of dislocation density for three nanowires under sequential electropulsing, compared within a unified coordinate system.**

**Figure S8. Repetitive observation of dislocation evolution in Pt nanocontact.** (a) Initial bent coherent nanocontact formed by welding two nanograins with a ~12° mismatch viewed along the [01$\bar{\text{1}}$] zone axis. The coherent {111} planes are marked by eight gradient-colored dotted lines as reference. A dislocation is located on the left surface. (b) A pulse drives dislocation climb. (c) A second pulse leads to dislocation annihilation with a new atomic layer (indicated by red “x”) formation.

**Note S1: Estimation of the temperature rise of nanowires under electropulsing**

Importantly, our nanowire is welded between two large metallic rods that acts as effective heat sinks. The dissipation time of Joule heating can be estimated by $\tau\approx\frac{{l_{\mathrm{diff}}}^{2}}{\alpha}$, where *l*_diff_ is the length of thermal diffusion (half of the nanowire length *l* in our work), and *α* is the thermal diffusivity of specimen, which is defined as $\alpha=\frac{\kappa}{\rho c_{p}}$, where κ is the thermal conductivity, ρ is the density and $c_{p}$ is the specific heat capacity. The typical values of these parameters for Mo are^[48]^: *κ*=138 W m^-1^ K^-1^, *ρ*=10280 kg m^-3^, *c*_p_=251 J kg^-1^ K^-1^. Substituting these values, we get α_Mo_=5.35×10^-5^ m^2^ s^-1^. Given the *l*_diff_ is estimated to be ~10 nm (Fig. 1), the dissipation time is estimated to be ~2×10^-3^ ns, ensuring that heat generated during the pulse can be efficiently conducted away before significant temperature accumulation occurs. Therefore, a steady-state one-dimensional heat conduction model is adopted to estimate the temperature rise^[49]^:

$$\Delta T_{max}=\frac{j^{2}\rho_{e}l^{2}}{8\kappa}$$

where *j* is the current density, *ρ*_e_, *l* and *κ* are the electrical resistivity, length and thermal conductivity of the specimen, respectively. Given that the estimated current density j~10^9^ A/cm^2^ in our experiment, the electrical resistivity of Mo at room temperature is ~5.3×10^-8^ Ω m, *κ*~138 W m^-1^ K^-1^, and *l*~20 nm, the maximum temperature rise of Mo nanowire is ~2 K. Considering the size effect of the nanowires, $\frac{\rho_{e}}{\kappa}$ can be approximately increased by one order of magnitude^[50]^. Nonetheless, such a low temperature rise is far below the temperature required for thermal activation of dislocation nucleation in Mo. Therefore, pure Joule heating alone cannot account for the observed dislocation nucleation and evolution.

**Note S2: Dislocation evolution under prolonged electropulsing**

To research the long-term evolution of dislocations, we performed prolonged electropulsing (200 sequential pulses, 2.5 V) experiment on a Mo nanowire, as shown in Figure S5.

Overall, the trend of dislocation number shows a general decrease during the 200-pulse sequence. This indicates that the system gradually evolves toward a lower-defect state. However, we still observe pulse-to-pulse fluctuations in dislocation count. This means periodic nucleation and annihilation events continue to occur, consistent with the behavior shown in Figure 2a for the first 20 pulses. This suggests that the local dynamic equilibrium between dislocation nucleation and annihilation persists even after many pulses.

The downward trend in dislocation density can be attributed to the consumption of nucleation sources and the relaxation toward a stable configuration. Structural heterogeneities (vacancies and surface steps) that serve as preferential nucleation sites become gradually exhausted or passivated after repeated pulsing and the system tends to a lower-energy, structurally more relaxed state after extended pulsing.

**Note S3: Discussions on electropulse voltages**

In our in-situ nanowelding approach, the nanowire dimensions are coupled to the welding voltage. After nanowire formation, applying excessively high voltage leads to premature melting or fracture, while too low voltage fails to provide sufficient driving force for dislocation motion.

We changed the pulse voltages applying on the nanowire in Figure S4a after 90 pulses, as shown in Figure S6. Pulse sequences were applied at three stages respectively: (i) 3.0 V for pulses 1–90; (ii) 2.6 V for pulses 91–120; (iii) 2.5 V for pulses 121–200. At the higher initial voltage (3.0 V), the fluctuations of dislocation number are more pronounced with larger amplitudes. As pulsing proceeds, the fluctuation amplitude gradually decreases, accompanied by a general downward trend in dislocation number. Upon switching to the lower voltage (2.6 V), the declining trend becomes more significant—the dislocation count decreases more rapidly and with reduced fluctuation amplitude. Further reduction to 2.5 V continues this trend, with few dislocations remaining and negligible nucleation events.

These observations suggest that higher voltage thus current density may promote more active dislocation nucleation (larger fluctuation amplitude), while lower current density favors net annihilation over nucleation, likely because the reduced electron wind force is insufficient to overcome the nucleation barrier at structural heterogeneities. The gradual decline in fluctuation amplitude even at constant voltage is attributed to the consumption of nucleation sources over extended pulsing.

**Reference**

[48]	C. L. Yaws, *Chemical properties handbook*, McGraw-Hill Companies, Inc, New York **1999**.

[49]	X. Li, J. Turner, K. Bustillo, A. M. Minor, *Acta Mater.* **2022**, *223*, 117461.

[50]	W. Ma, X. Zhang, *Int. J. Heat Mass Transfer* **2013**, *58* (1), 639.
